# Limited HIV-1 Reactivation in Resting CD4^+^ T cells from Aviremic Patients under Protease Inhibitors

**DOI:** 10.1038/srep38313

**Published:** 2016-12-06

**Authors:** Amit Kumar, Wasim Abbas, Sophie Bouchat, Jean-Stéphane Gatot, Sébastien Pasquereau, Kabamba Kabeya, Nathan Clumeck, Stéphane De Wit, Carine Van Lint, Georges Herbein

**Affiliations:** 1 Department of Virology, Pathogens & Inflammation Laboratory, University of Franche-Comté, COMUE Bourgogne Franche-Comté University, UPRES EA4266, SFR FED 4234, CHRU Besançon, France; 2 Laboratory of Molecular Virology, IBMM, Université Libre de Bruxelles (ULB), Gosselies, Belgium; 3 Department of Infectious Diseases, CHU St-Pierre, ULB, Bruxelles, Belgium

## Abstract

A latent viral reservoir that resides in resting CD4^+^ T cells represents a major barrier for eradication of HIV infection. We test here the impact of HIV protease inhibitor (PI) based combination anti-retroviral therapy (cART) over nonnucleoside reverse transcriptase inhibitor (NNRTI)-based cART on HIV-1 reactivation and integration in resting CD4^+^ T cells. This is a prospective cohort study of patients with chronic HIV-1 infection treated with conventional cART with an undetectable viremia. We performed a seven-year study of 47 patients with chronic HIV-infection treated with cART regimens and with undetectable plasma HIV-1 RNA levels for at least 1 year. Of these 47 patients treated with cART, 24 were treated with a PI-based regimen and 23 with a NNRTI-based regimen as their most recent treatment for more than one year. We evaluated the HIV-1 reservoir using reactivation assay and integrated HIV-1 DNA, respectively, in resting CD4^+^ T cells. Resting CD4^+^ T cells isolated from PI-treated patients compared to NNRTI-treated patients showed a limited HIV-1 reactivation upon T-cell stimulation (p = 0·024) and a lower level of HIV-1 integration (p = 0·024). Our study indicates that PI-based cART could be more efficient than NNRTI-based cART for limiting HIV-1 reactivation in aviremic chronically infected patients.

Among the main features of human immunodeficiency virus-1 (HIV-1) infection are immune suppression and viral persistence^1^. Combination anti-retroviral therapy (cART) drives the viral load down to undetectable levels^2^. However, using the ultrasensitive assays, low levels of active viral replication can be detected in the majority of subjects successfully treated with cART^3^. Indeed, the persistence of latent reservoirs of replication-competent proviruses remains a major obstacle in HIV-1 eradication^4^,^5^. Latent reservoirs are established early during acute viral infection and include among others, macrophages and latently infected resting CD4^+^ T cells, these later being the main viral reservoir^6^,^7^,^8^.

Most of the studies so far have addressed the effect of cART on the decrease of HIV-1 viremia under limit of detection using the classical assays. Usually, a better virologic performance of nonnucleoside reverse transcriptase inhibitor-(NNRTI)-based cART compared to protease inhibitor-(PI)-including regimens has been reported^9^. Although PI-based regimen have lower rates of HIV suppression compared with the NNRTI-based treatments, greater CD4 cell increases are seen in patients on PI arm and there is also less development of major drug resistance mutations in patients failing PI-based therapy^10^,^11^. Recently, cART intensification was assessed and did not reduce residual HIV-1 viremia in patients on cART, indicating that its potential to eradicate the virus appears limited^12^.

In contrast to the measurement of viremia in patients on cART, the impact of cART on the size of the cellular reservoirs of HIV-1 has been much less studied. Initiation of cART during primary HIV infection may limit the establishment of viral reservoirs, and very early cART limits the seeding of the HIV reservoir in long-lived central memory CD4^+^ T cells^6^,^13^,^14^. By contrast, the impact of cART on the HIV reservoir especially on viral reactivation from resting CD4^+^ T cells in aviremic chronically infected patients is so far unknown. We report here a study indicating a higher efficiency of PI-based cART over NNRTI-based cART for limiting HIV-1 reactivation in CD4^+^ T cells from aviremic chronically HIV-1 infected patients.

## Results

Forty-seven patients with chronic HIV-1 infection treated with cART (treatment range: 2 years to 16 years) and with undetectable plasma HIV-1 RNA levels (<40 copies/ml) for at least 1 year were included in the study between 2008 and 2014. Of these 47 patients (mean age 47.4 years; range 27–93 years) treated with cART, 24 were treated with PI-based cART and 23 with NNRTI-based cART (as their most recent treatment) for more than one year (Table 1, Supplementary Tables S1 and S2). We did not observe significant differences for nadir median CD4 counts (298·10^6^ versus 333·10^6^ cells/l, *P* = 0.579), median CD4 counts at initiation of treatment (384·10^6^ versus 349·10^6^ cells/l, *P* = 0·903), median CD4 counts at the last point (607·10^6^ versus 624·10^6^ cells/l, *P* = 0·647), and median HIV RNA load at zenith (4·92 versus 4·83 log/ml, *P* = 0·892) between PI-treated and NNRTI-treated patients (Table 1). We did not find significant differences between PI-treated and NNRTI-treated patients for the number of failures of treatment (1 versus 1, *P* = 0·985) and the duration of therapy (8·50 versus 8·52 years, *P* = 0·936). The duration with undetectable plasma HIV-1 RNA levels was not significantly different in the PI-treated arm compared to the NNRTI-treated arm (4·83 versus 5·39 years, *P* = 0·581) (Table 1). At the last treatment, in 51·1% of individuals cART included a PI (fosamprenavir; saquinavir; atazanavir; lopinavir/ritonavir; darunavir) and in 48·9% a NNRTI (nevirapine; efavirenz; rilpivirine). Of note, among the 47 patients treated with cART, 17 were treated only with PI and 7 only with NNRTI (Supplementary Tables S1 and S2).

**Figure 1:**
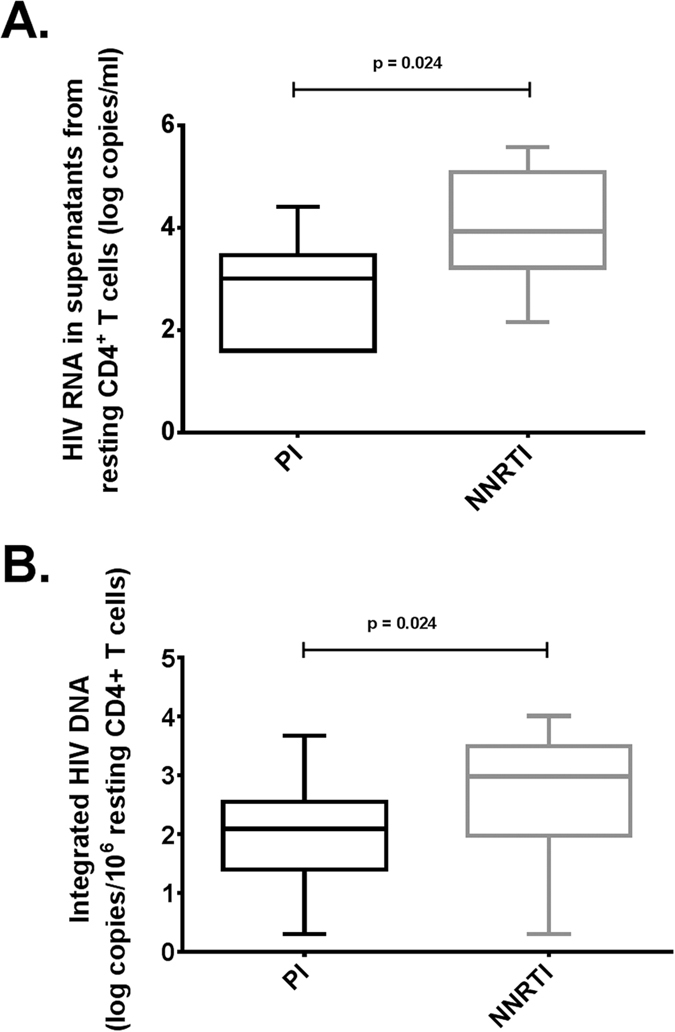
Resting CD4^+^ T cells isolated from PI-treated patients compared to NNRTI-treated patients showed a limited HIV-1 reactivation upon T-cell stimulation and a lower level of HIV-1 integration. (**A**) Comparison of reactivation assay on resting CD4^+^ T cells isolated from HIV^+^ patients with undetectable viremia treated with PI (n = 10) and NNRTI (n = 11) as their lastly administered treatment. (**B**) Comparison of measurement of integrated HIV-1 DNA in resting CD4^+^ T cells isolated from HIV^+^ patients with undetectable viremia treated with PI (n = 14) and NNRTI (n = 12) as their lastly administered treatment.

In order to evaluate the qualitative and quantitative patient-specific variations in the *ex vivo* reactivation capacity of the HIV-infected cells depending of cART treatment, we analyzed the effect of a known HIV inducer (anti-CD2+anti-CD28 antibodies)^15^,^16^ on viral reactivation in *ex vivo* cultures of purified resting CD4^+^ T cells isolated from HIV^+^ patients treated with PI-based cART and with NNRTI-based cART. Since latently infected resting CD4^+^ T cells that harbour integrated replication-competent viral DNA represent the primary long-lived source of persistent HIV-1 in patients under cART^5^,^6^, we decided to analyze the impact of cART regimen on resting CD4^+^ T cells isolated from HIV^+^ patients. We observed, following reactivation of HIV-1 from latency in purified resting CD4^+^ T cells, that HIV-1 recovery was 0·92 log of HIV RNA copies/ml (3·01 *versus* 3·93 log copies/ml, *P* = 0·024) lower in HIV-1-infected patients treated with PI compared to those treated with NNRTI as their lastly administered treatment (Tables 1 and 2, and Fig. 1A). The impact of PI versus NNRTI treatment, taking into account the last treatment administered, on latently infected CD4^+^ T cells was further assessed by quantifying integrated HIV-1 DNA. The levels of integrated HIV-1 DNA was 0·89 log (2·09 *versus* 2·98 log copies/10^6^ cells, *P* = 0·024) lower in purified resting CD4^+^ T cells isolated from HIV-1 patients treated with PI-based cART compared to HIV-1-infected patients treated with NNRTI-based cART (Tables 1 and 3, and Fig. 1B).

## Discussion

To diminish the size of the viral reservoir, several studies have shown that an early treatment during HIV-1 primo-infection could be beneficial^13^,^14^, and recently on-demand preexposure prohylaxis has been shown to be highly efficient to prevent HIV transmission^17^. We report here a study indicating that PI-based cART could be more efficient than NNRTI-based cART for limiting HIV-1 reactivation in aviremic chronically infected patients.

Although the respective use of NNRTI-based cART and PI-based cART have been extensively studied in regard to residual viremia, only little is known on their impact on cellular reservoirs of HIV-1. By two approaches, reactivation assay and the Alu-PCR assay that allow evaluating in a qualitative and quantitative manner, respectively, the amount of integrated provirus, we observed that PI-based cART was superior to NNRTI-based cART for limiting the size of the HIV-1 reservoir. Recent studies indicate that raltegravir intensification of PI-based cART results in a specific and transient increase in episomal HIV DNA that might represent lower efficiency of HIV integration under PI^18^,^19^. Recent publications also indicate that the size of the HIV-1 reservoir measured by total cell-associated HIV-1 DNA correlates positively with the frequency of positive recovery measurements in response to various latency-reversing agents^20^. Additionally, the low frequency of infected cells in the HIV-1 controllers is associated with less efficient viral reactivation^21^. Our data are in line with these findings indicating that limited reactivation could be a consequence of lower integration events.

Several molecular mechanisms could explain the lower viral reactivation under PIs. First, low frequency of viral integration in transcriptionally active sites could explain the limited reactivation observed under PIs. Second, since integrase is produced by protease-mediated cleavage of the Pr160^Gag-Pol^ precursor protein, PIs might inhibit HIV-1 integration by preventing the formation of a functional integrase^19^. Impaired integrase activity and/or impairment of the integrase-LEDGF/p75 interaction could result in a decreased number of integrated provirus in transcriptionally active sites, but also could favor the viral integration in less transcriptionally active sites which will result in lower viral reactivation^22^. Third, HIV PIs have been reported to block Akt activation^23^, and we recently observed that PIs, by blocking Akt activation especially triggered by Nef, limit HIV-1 recovery from latently-infected T cells^24^. Additionally HIV integration and the establishment of latency in CCL19-treated resting CD4^+^ T cells has been shown to require activation of NF-kB and increased HIV integrase stability^25^. Interestingly, PIs block NF-kB activation induced by Toll-like receptor 2 (TLR2) and TLR4^26^. We also observed that PIs, but not NNRTIs, block Akt activation in PBLs treated with recombinant HIV-1 Nef *in vitro* and that the blockade of Nef-mediated Akt activation by Akt inhibitors decreased dramatically NF-kB activation^24^. Therefore, we cannot exclude that PIs by blocking HIV-mediated Akt activation in T cells decrease NF-kB activation and ultimately limit HIV integration in T cells.

Since Akt activation is critical for cell survival, the blockade of Akt activation by PIs in latently infected resting CD4^+^ T cells could help to clear these cells through accelerated cell death. Additionally, PIs have been shown to trigger the death of CD4^+^ T cells through the generation of Casp8p41, a fragment obtained by cleavage of procaspase 8 by PIs, that lacks caspase activity but nonetheless contributes to T cell apoptosis^27^. Thus PIs could participate to decrease the size of the viral reservoir in infected patients through the clearance of infected CD4^+^ T cells.

Low HIV-1 reservoirs found in HIV-1 controllers contributes to temporary control of infection without antiretroviral therapy^21^. Along this line, control of HIV-1 viremia or delayed viral rebound after discontinuation of antiretroviral treatment has been consistently associated with low levels of cell associated HIV-DNA at the time of treatment interruption^28^,^29^. Therefore, lower levels of integrated HIV-1 in purified resting CD4^+^ T cells isolated from HIV-1 patients treated with PI-based cART compared to HIV-1-infected patients treated with NNRTI-based cART could be critical to achieve control of infection.

In conclusion, in aviremic chronically HIV-1-infected patients our study indicates that PI-based cART limits HIV-1 recovery from latently infected resting CD4^+^ T cells and curtails the amount of integrated HIV-1 DNA. Altogether, our results support the need to further assess the therapeutic use of HIV-1 protease inhibitors in view of their impact on persistence of HIV-1 reservoirs and might provide and/or accompany new therapeutic strategies for a remission.

## Methods

### Ethics approval and consent to participate

All the patients who were enrolled at the St-Pierre University Hospital (Brussels, Belgium) and the Besançon University Hospital (France) gave their written informed consent to participate in the study according to the Helsinki declaration. The study was approved by the local ethics committees of the Saint-Pierre University Hospital (Brussels, Belgium) and the Besancon University Hospital (Besançon, France) (CPP EST-2). To perform our study, we used blood samples that were part of a routine care. It was an observational study and not a clinical trial. According to the French Regulatory Authority for clinical studies, prospective and retrospective studies with observation analysis only are not evaluated by Human Protection Committees. The Human Protection Committee East Area II from France was consulted and issued a formal waiver of approval. This study did not rely solely on medical records. The authors did not have any contact with the study subjects and performed tests on patient blood samples that were part of a routine care. The blood samples were anonymized before being used by the authors.

### Patients

We enrolled 47 chronically HIV-1 infected individuals at the St-Pierre Hospital (Brussels, Belgium) and the Besançon University Hospital (Besançon, France). The patients were treated with cART (treatment range: 2 years to 16 years) and had undetectable plasma HIV-1 RNA levels (<40 copies/ml) for at least 1 year. Characteristics of these patients were well documented and presented in Supplementary Tables S1 and S2. Of these 47 patients treated with cART, 24 were treated with PI and 23 with NNRTI as their most recent treatment for more than one year.

### Isolation of resting CD4^+^ T lymphocytes

Purified CD25^−^, CD69^−^, HLA-DR^−^ CD4^+^ T cells (resting CD4^+^ T cells) were obtained from the peripheral blood of HIV^+^ patients using a negative selection assay using magnetic beads (LD columns, Miltenyi Biotec, Bergisch Gladbach, Germany) as described previously^30^.

### Viral reactivation assay

One day after isolation, 5 × 10^5^ purified CD25^−^, CD69^−^, HLA-DR^−^ CD4^+^ T cells (resting CD4^+^ T cells) were mock-treated or treated with anti-CD2+ anti-CD28 antibodies as a positive control for global T-cell activation^15^,^16^. For the measurement of reactivation of HIV-1 expression in resting CD4^+^ T cells isolated from the peripheral blood of HIV-infected patients, six days after treatment, culture supernatants were tested for quantitative HIV-1 RNA levels using the COBAS AmpliPrep/COBAS Amplicor HIV-1 Monitor Test, according to the manufacturer’s instructions.

### Quantification of integrated HIV-1 DNA

Integrated HIV-1 DNA was measured by using a previously described *Alu-gag* PCR with some modifications^31^. This assay detects only integrated provirus because it relies on initial amplification in which one primer hybridizes with conserved sequences in *Alu* elements that are present in the human genome. The level of HIV-1 integration was measured by comparing the detection signal to an integration standard curve that was prepared from U1 cells. ACH2 cells were used for calibration. The sequences of first step PCR amplification primers were as follows: genomic *Alu* forward, 5′-GCCTCCCAAAGTGCTGGGATTACAG-3′; and HIV-1 gag reverse, 5′-GCTCTCGCACCCATCTCTCTCC-3′. The reaction conditions were 1x Tfi PCR reaction buffer, 1.5 mM MgCl_2_, 1 mM dNTPs, 100 nM *Alu* forward primer, 600 nM gag reverse primer and 5 U of Tfi DNA polymerase (Invitrogen). The thermal cycler (Eppendorf AG, Hamburg, Germany) was programmed to perform a 2 min hot start at 94 °C, followed by 20 cycles of following: denaturation at 94 °C for 30 sec, annealing at 50 °C for 1 min, and extension at 72 °C for 1 min 40 sec. The second round of real time quantitative PCR was performed using 21 μl of Nested Alu-gag PCR along with standard curve amplification products. The sequences of the primers were as follows: LTR forward, 5′-GCCTCAATAAAGCTTGCCTTGA-3′; and LTR reverse, 5′-TCCACACTGACTAAAAGGGTCTGA-3′. The LTR Taqman probe, labeled at its 5′ terminus with the reporter fluorophore 6-carboxyfluorescein (FAM) and at its 3′ terminus with the quencher 4-(4-dimethylamino-phenylazo)-benzene (DABCYL), had the following sequence: 5′-FAM-GCGAGTGCCCGTCTGTTGTGTGACTCTGGTAACTAGCTCGC-DABCYL-3′. The reaction was carried out in a volume of 50 μl containing 1 X Taqman Master mix (Applied Biosystems, Foster City, CA), 250 nM concentration of LTR forward and reverse primers, and 200 nM Taqman probe. The reactions were performed on Stratagene Mx2005 *P*. The thermal program was 2 min hot start at 95 °C, followed by 40 cycles of denaturation at 95 °C for 15 sec and annealing and extension at 60 °C for 1 min.

### Data analysis

The program used for plotting and statistical analysis was Prism version 6.0 (GraphPad Software). Results from e*x vivo* reactivation studies and HIV integration using patient cell cultures of resting CD4^+^ T cells are shown as medians. Data sets were analyzed using an unpaired nonparametric t-test (Mann-Whitney test). Differences were considered significant at a value of p < 0.05.

## Additional Information

**How to cite this article**: Kumar, A. *et al*. Limited HIV-1 Reactivation in Resting CD4^+^ T cells from Aviremic Patients under Protease Inhibitors. *Sci. Rep.* **6**, 38313; doi: 10.1038/srep38313 (2016).

**Publisher's note:** Springer Nature remains neutral with regard to jurisdictional claims in published maps and institutional affiliations.

## Supplementary Material

Supplementary Information

## Figures and Tables

**Table 1:** Comparison of biological and virological characteristics of HIV^+^ patient groups under PI-cART and NNRTI-cART.

*Biological characteristics*	PI	NNRTI	*P value*
	*(n* = *24)*	*(n* = *23)*	
Age (years), mean (±s.d.)	48.6 ± 12.7	46.2 ± 10.7	*0.495*
CD4^+^ T cell counts at nadir, absolute (cells per μl), median (IQR)	298 (201–375)	333 (246–419)	*0.579*
CD4^+^ T cell counts at initiation of treatment, absolute (cells per μl), median (IQR)	384 (285–461)	349 (265–574)	*0.903*
CD4^+^ T cell counts at the last point, absolute (cells per μl), median (IQR)	607 (480–832)	624 (492–801)	*0.647*
pVL at zenith (log copies per ml), median (IQR)	4.92 (4.23–5.07)	4.83 (4.47–5.16)	*0.892*
Previous treatment failure, median (IQR)	1·00 (0–2.25)	1·00 (0–4)	*0.985*
Time with therapy (years), mean ± s.d.	8.50 ± 3.63	8.52 ± 3.49	*0.936*
Time with undetectable pVL (years), mean ± s.d.	4.83 ± 2.97	5.39 ± 2.73	*0.581*
***HIV-1 reactivation assay***			
** Resting CD4**^**+**^**T cells**	***(n*** = ***10)***	***(n*** = ***11)***	
** **HIV-1 RNA levels after **reactivation assay** (log copies per ml), median (IQR)	3.01 (1.84–3.45)	3.93 (3.31–4.87)	0.024*
***HIV-1 integration assay***			
** Resting CD4**^**+**^ **T cells**	***(n*** = ***14)***	***(n*** = ***12)***	
** Integrated** HIV-1 DNA (log copies per 10^6^ cells), median (IQR)	2.09 (1.53–2.52)	2.98 (2.55–3.48)	0.024*

IQR, interquartile range; pVL, plasma viral load *P < 0·05.

**Table 2:** Reactivation assay on HLA DR^−^ CD25^−^ CD69^−^ CD4^+^ T cells (resting CD4^+^ T cells) isolated from HIV^+^ patients with undetectable viremia treated with PI (n = 10) and NNRTI (n = 11) as their lastly administered treatment.

Patients	CD4+ at last point (cells/microl)	Last treatment	Duration of therapy (years)	Duration with undetectable plasma HIV-1 RNA levels (years)	HIV-1 RNA levels after reactivation assay (copies/ml)	HIV-1 RNA levels after reactivation assay (log copies/ml)
**PI at the last treatment (n** = **10)**						
WSH 229	617	ABC 3TC SQV	13	12	25862	4.41
WSH 231	770	ABC 3TC ATV	10	7	40	1.60
WSH 232	899	TDF FTC fAPV	7	1	3106	3.49
WSH 233	465	ATV EFV	8	8	40	1.60
WSH 235	797	DDI AZT 3TC fAPV	15	5	1108	3.04
WSH 236	402	TDF FTC ATV	13	1	2862	3.46
WSH 238	1240	TDF FTC ATV	5	5	40	1.60
WSH 239	999	TDF FTC ATV	9	1	954	2.98
WSH 240	663	TDF FTC ATV	9	4	2548	3.41
WSH 241	524	TDF FTC fAPV	2	2	372	2.57
**Mean**	**738**		**9.10**	**4.60**	**3693**	**2.82**
**SD**	**246**		**3.73**	**3.44**	**7477**	**0.91**
**Median**	**717**		**9.00**	**4.50**	**1031**	**3.01**
**1st Quartile**	**547**		**7.25**	**1.25**	**123**	**1.84**
**3rd Quartile**	**874**		**12.3**	**6.50**	**2784**	**3.45**
**NNRTI at the last treatment (n** = **11)**						
WSH 227	733	EFV FTC TDF	9	8	146	2.16
WSH 228	1324	TDF FTC NVP	10	7	229674	5.36
WSH 230	444	EFV FTC TDF	8	1	8492	3.93
WSH 242	428	DDI TDF ABC 3TC EFV	10	3	1668	3.22
WSH 243	794	EFV FTC TDF	6	1	2514	3.40
WSH 244	479	EFV FTC TDF	8	8	122268	5.09
WSH 245	478	TDF FTC NVP	12	3	43614	4.64
WSH 246	629	EFV FTC TDF	3	1	374578	5.57
WSH 247	498	ABC 3TC NVP	16	7	31976	4.50
WSH 248	1017	ABC 3TC EFV	10	7	4224	3.63
WSH 249	519	AZT 3TC NVP	13	11	668	2.82
**Mean**	**668**		**9.55**	**5.18**	**74529**	**4.03**
**SD**	**270**		**3.31**	**3.33**	**116748**	**1.05**
**Median**	**519**		**10.0**	**7.00**	**8492**	**3.93**
**1st Quartile**	**479**		**8.00**	**2.00**	**2091**	**3.31**
**3rd Quartile**	**764**		**11.0**	**7.50**	**82941**	**4.87**
**MW p-value**	**0.461**		**0.768**	**0.684**	**0.024**	**0.024**

**Table 3:** Measurement of integrated HIV-1 DNA in HLA DR^−^ CD25^−^ CD69^−^ CD4^+^ T cells (resting CD4^+^ T cells) isolated from HIV^+^ patients with undetectable viremia treated with PI (n = 14) and NNRTI (n = 12) as their lastly administered treatment.

Patients	CD4+ at the last point (cells/microl)	Last treatment	Duration of therapy (years)	Duration with undetectable plasma HIV-1 RNA levels (years)	Integrated HIV-1 DNA (copies/10^6 cells)	Integrated HIV-1 DNA (log copies/10^6 cells)
**PI at the last treatment (n** = **14)**						
B9	485	TDF FTC ATV	11	8	95	1.97
B13	512	ABC 3TC ATV	14	8	85	1.92
B15	995	TDF FTC ATV	12	8	4731	3.67
B27	529	ABC 3TC ATV	3	2	4	0.6
B33	404	TDF FTC DRV	8	2	175	2.24
B34	127	TDF FTC DRV	2	1	17	1.23
B38	423	TDF FTC ATV	8	5	2	0.3
B39	997	ABC 3TC DRV	8	3	409	2.61
B40	394	fAPV AZT 3TC	8	5	56	1.74
B41	596	TDF FTC DRV	3	2	29	1.46
B42	638	DRV TDF ABC 3TC	8	5	307	2.48
B43	505	ABC 3TC ATV	10	8	163	2.21
B45	809	ABC TDF DRV	12	8	344	2.53
B46	906	ABC 3TC ATV	6	5	635	2.8
**Mean**	**594**		**8.07**	**5.00**	**504**	**1.98**
**SD**	**242**		**3.49**	**2.56**	**1186**	**0.85**
**Median**	**521**		**8.00**	**5.00**	**129**	**2.09**
**1st Quartile**	**439**		**6.50**	**2.25**	**35.8**	**1.53**
**3rd Quartile**	**766**		**10.8**	**8.00**	**335**	**2.52**
**NNRTI at the last treatment (n** = **12)**						
B2	548	EFV FTC TDF	5	4	6174	3.79
B3	927	ABC 3TC NVP	7	5	2	0.3
B6	479	NVP TDF FTC	8	7	682	2.83
B8	975	EFV FTC TDF	3	2	985	2.99
B10	580	EFV TDF FTC	6	5	2963	3.47
B14	486	NVP TDF FTC	5	4	944	2.97
B16	928	TDF FTC NVP	15	8	2879	3.46
B18	624	TDF FTC RPV	5	4	10218	4.01
B21	607	TDF FTC RPV	12	8	49	1.69
B22	793	TDF FTC NVP	5	4	38	1.58
B29	807	TDF FTC RPV	10	8	3185	3.5
B37	790	TDF FTC RPV	10	8	771	2.88
**Mean**	**712**		**7.58**	**5.58**	**2408**	**2.79**
**SD**	**171**		**3.38**	**2.02**	**2931**	**1.04**
**Median**	**707**		**6.50**	**5.00**	**965**	**2.98**
**1st Quartile**	**572**		**5.00**	**4.00**	**524**	**2.55**
**3rd Quartile**	**837**		**10.0**	**8.00**	**3019**	**3.48**
**MW p-value**	**0.210**		**0.583**	**0.719**	**0.024**	**0.024**

## References

[b1] Chun T. W., Moir S. & Fauci A. S. HIV reservoirs as obstacles and opportunities for an HIV cure. Nat. Immunol. 16, 584–589 (2015). 25990814 10.1038/ni.3152

[b2] Perelson A. S. . Decay characteristics of HIV-1-infected compartments during combination therapy. Nature 387, 188–191 (1997). 9144290 10.1038/387188a0

[b3] Palmer S. . Low-level viremia persists for at least 7 years in patients on suppressive antiretroviral therapy. Proc. Natl. Acad. Sci. USA. 105, 3879–3884 (2008). 18332425 10.1073/pnas.0800050105 PMC2268833

[b4] Ho Y. C. . Replication-competent noninduced proviruses in the latent reservoir increase barrier to HIV-1 cure. Cell 155, 540–551 (2013). 24243014 10.1016/j.cell.2013.09.020 PMC3896327

[b5] Van Lint C., Bouchat S. & Marcello A. HIV-1 transcription and latency: an update. Retrovirology 10, 67 (2013). 23803414 10.1186/1742-4690-10-67 PMC3699421

[b6] Siliciano R. F. Opening fronts in HIV vaccine development: targeting reservoirs to clear and cure. Nat. Med. 20, 480–481 (2014). 24804757 10.1038/nm.3550

[b7] Le Douce V. . Molecular mechanisms of HIV-1 persistence in the monocyte-macrophage lineage. Retrovirology 7, 32 (2010). 20380694 10.1186/1742-4690-7-32 PMC2873506

[b8] Kumar A., Darcis G., Van Lint C. & Herbein G. Epigenetic control of HIV-1 post integration latency: implications for therapy. Clin. Epigenetics 7, 103 (2015). 26405463 10.1186/s13148-015-0137-6 PMC4581042

[b9] INITIO Trial International Co-ordinating Committee . Virological and immunological outcomes at 3 years after starting antiretroviral therapy with regimens containing non-nucleoside reverse transcriptase inhibitor, protease inhibitor, or both in INITIO: open-label randomised trial. Lancet 368, 287–298 (2006). 16860698 10.1016/S0140-6736(06)69074-0

[b10] Cummins N. W., Sainski A. M., Natesampillai S., Bren G. D. & Badley A. D. Choice of antiretroviral therapy differentially impacts survival of HIV-infected CD4 T cells. Mol. Cell. Ther. 2, 1 (2014). 10.1186/2052-8426-2-1 PMC4448955 26057236

[b11] Chéret A. . Combined ART started during acute HIV infection protects central memory CD4^+^ T cells and can induce remission. J. Antimicrob. Chemother. 70, 2108–2120 (2015). 25900157 10.1093/jac/dkv084

[b12] Dinoso J. B. . Treatment intensification does not reduce residual HIV-1 viremia in patients on highly active antiretroviral therapy. Proc. Natl. Acad. Sci. USA. 106, 9403–9408 (2009). 19470482 10.1073/pnas.0903107106 PMC2685743

[b13] Sáez-Cirión A. . Post-treatment HIV-1 controllers with a long-term virological remission after the interruption of early initiated antiretroviral therapy ANRS VISCONTI study. Plos Pathog. 9, e1003211 (2013). 23516360 10.1371/journal.ppat.1003211 PMC3597518

[b14] Buzón M. J. . HIV-1 replication and immune dynamics are affected by raltegravir intensification of HAART-suppressed subjects. Nat. Med. 16, 460–465 (2010). 20228817 10.1038/nm.2111

[b15] Costello R. . Activation of primary human T-lymphocytes through CD2 plus CD28 adhesion molecules induces long-term nuclear expression of NF-kappa B. Cell Growth Differ. 4, 329–339 (1993). 8098618

[b16] Pierrès A. . Triggering CD28 molecules synergize with CD 2 (T 11.1 and T 11.2)-mediated T cell activation. Eur. J. Immunol. 18, 685–690 (1988). 2454190 10.1002/eji.1830180505

[b17] Molina J. M. . On-demand preexposure prophylaxis in men at high risk for HIV-1 infection. N. Engl. J. Med. 373, 2237–2246 (2015). 26624850 10.1056/NEJMoa1506273

[b18] Hatano H. . Increase in 2-long terminal repeat circles and decrease in D-dimer after raltegravir intensification in patients with treated HIV infection: a randomized, placebo-controlled trial. J. Infect. Dis. 208, 1436–1442 (2013). 23975885 10.1093/infdis/jit453 PMC3789577

[b19] Rabi S. A. . Multi-step inhibition explains HIV-1 protease inhibitor pharmacodynamics and resistance. J. Clin. Invest. 123, 3848–3860 (2013). 23979165 10.1172/JCI67399 PMC4381280

[b20] Darcis G. . Reactivation capacity by latency-reversing agents correlates with the size of the HIV-1 reservoir. AIDS PMID: 27755105 (2016). 10.1097/QAD.0000000000001290 27755105

[b21] Noel N. . Long-term spontaneous control of HIV-1 relates to low frequency of infected cells and inefficient viral reactivation. J. Virol. 90, 6148–6158 (2016). 27122576 10.1128/JVI.00419-16 PMC4907242

[b22] Vranckx L. S. . LEDGIN-mediated inhibition of integrase-LEDGF/p75 interaction reduces reactivation of residual latent HIV. EBioMedicine 8, 248–264 (2016). 27428435 10.1016/j.ebiom.2016.04.039 PMC4919729

[b23] Kourjian G. . HIV protease inhibitor-induced cathepsin modulation alters antigen processing and cross-presentation. J. Immunol. 196, 3595–3607 (2016). 27009491 10.4049/jimmunol.1600055 PMC4868670

[b24] Kumar A. . Fine tuning of AKT-pathway by Nef and its blockade by protease inhibitors results in limited recovery in latently HIV infected T-cell line. Sci Rep. 6, 24090 (2016). 27076174 10.1038/srep24090 PMC4831010

[b25] Saleh S. . HIV integration and the establishment of latency in CCL19-treated resting CD4^+^ T cells require activation of NF-kB. Retrovirology 13, 49 (2016). 27459960 10.1186/s12977-016-0284-7 PMC4962537

[b26] Equils O. . Human immunodeficiency virus type 1 protease inhibitors block Toll-like receptor 2 (TLR2)- and TLR4-induced NF-kappaB activation. Antimicrob. Agents Chemother. 48, 3905–3911 (2004). 15388451 10.1128/AAC.48.10.3905-3911.2004 PMC521905

[b27] Sainski A. M. . Casp8p41 generated by HIV protease kill CD4 T cells through direct Bak activation. J Cell Biol 206, 867–876 (2014). 25246614 10.1083/jcb.201405051 PMC4178959

[b28] Assoumou L. . A low HIV-DNA level in peripheral blood mononuclear cells at antiretroviral treatment interruption predicts a higher probability of maintaining viral control. AIDS 29, 2003–2007 (2015). 26355572 10.1097/QAD.0000000000000734

[b29] Goujard C. . HIV-1 control after transient antiretroviral treatment initiated in primary infection: role of patient characteristics and effect of therapy. Antivir. Ther. 17, 1001–1009 (2012). 22865544 10.3851/IMP2273

[b30] Bouchat S. . Histone methyltransferase inhibitors induce HIV-1 recovery in resting CD4(+) T cells from HIV-1-infected HAART-treated patients. AIDS 26, 1473–1482 (2012). 22555163 10.1097/QAD.0b013e32835535f5

[b31] Liszewski M. K., Yu J. J. & O’Doherty U. Detecting HIV-1 integration by repetitive-sampling Alu-gag PCR. Methods 47, 254–260 (2009). 19195495 10.1016/j.ymeth.2009.01.002 PMC2862469

