# Supplementary material for: Limited HIV-1 Reactivation in Resting CD4+ T cells from Aviremic Patients under Protease Inhibitors
Source: Sci Rep. 2016 Dec 6;6:38313. doi: 10.1038/srep38313 (PMC5138822; doi:10.1038/srep38313)
Supplement: Supplementary Information [file srep38313-s1.pdf]

Limited HIV-1 Reactivation in Resting CD4$^+$ T cells from Aviremic Patients under Protease Inhibitors

Amit Kumar[1+], Wasim Abbas[1+], Sophie Bouchat[2], Jean-Stéphane Gatot[2,$], Sébastien Pasquereau[1], Kabamba Kabeya[3], Nathan Clumeck[3], Stéphane De Wit[3], Carine Van Lint[2++*], Georges Herbein[1++*]

[+] AK and WA, [++] GH and CVL contributed equally to the work.

[1] Department of Virology, Pathogens & Inflammation Laboratory, University of Franche-Comté, COMUE Bourgogne Franche-Comté University, UPRES EA4266, SFR FED 4234, CHRU Besançon, Besançon, France, [2] Laboratory of Molecular Virology, IBMM, Université Libre de Bruxelles (ULB), Gosselies, Belgium, [3] Department of Infectious Diseases, CHU St-Pierre, ULB, Bruxelles, Belgium, [$] Present address: Service de Génétique, Centre Hospitalier Universitaire (CHU) de Liège, Domaine Universitaire du Sart-Tilman, B23, 4000 Liège, Belgium.

* Correspondence to: Dr Georges Herbein, Department of Virology, University of Franche-Comté, CHRU Besançon, Hôpital Saint-Jacques, 2 place Saint-Jacques, F-25030 Besançon cedex, France Ph. +33 381 218 877; Fax + 33 381 665 695; E-mail: georges.herbein@univ-fcomte.fr Or Dr Carine Van Lint, Laboratory of Molecular Virology, Institut de Biologie et de Médecine Moléculaires (IBMM), Université Libre de Bruxelles (ULB), rue des Profs Jeener et Brachet 12, 6041 Gosselies, Belgium. Ph. +32 2650 98 07; Fax + 32 2650 98 00; E-mail: cvlint@ulb.ac.be

Supplementary Table S1: Biological and virological characteristics of HIV+ patient group under PI-cART taking into account the last treatment administered (n=24).

Patients	Age (years)	CD4+ Nadir (cells/microl)	CD4+ at initiation of treatment (cells/microl)	HIV RNA Zenith (copies/ml)	HIV RNA Zenith (log copies/ml)	First treatment	PI in the first treatment	Failure of treatment (number)	Always under PI	At least once under PI
**PI at the last treatment (n=24)**										
WSH 229	52	204	391	100000	5.00	D4T 3TC NFV	X	1	X	X
WSH 231	53	192	248	123000	5.09	D4T 3TC ATV	X	3	X	X
WSH 232	47	181	181	100000	5.00	AZT 3TC IDV	X	2	X	X
WSH 233	55	48	48	41800	4.62	AZT 3TC IDV	X	2	X	X
WSH 235	51	339	383	/	/	AZT DDC	0	4	0	X
WSH 236	43	153	226	285237	5.46	AZT DDI	0	9	0	X
WSH 238	46	749	749	100000	5.00	TDF FTC LPV	X	2	X	X
WSH 239	34	370	480	23871	4.38	3TC ABC NFV	X	5	0	X
WSH 240	41	568	568	/	/	AZT 3TC	0	4	0	X
WSH 241	37	339	385	789	2.90	3TC TDF fAPV	X	1	X	X
B9	54	284	342	58000	4.76	3TC TDF ATV	X	0	X	X
B13	93	391	424	115943	5.06	AZT 3TC fAPV	X	0	X	X
B15	49	735	800	11000	4.04	3TC NVP LPV	X	0	X	X
B27	51	312	397	6641	3.82	TDF FTC ATV	X	0	X	X
B33	50	118	341	74600	4.87	AZT 3TC EFV	0	4	0	X
B34	34	3	3	333071	5.52	TDF FTC DRV	X	0	X	X
B38	52	257	399	94000	4.97	TDF LPV 3TC	X	2	X	X
B39	22	252	500	36400	4.56	ABC LPV 3TC	X	2	X	X
B40	65	228	269	270000	5.43	AZT 3TC fAPV	X	1	X	X
B41	47	370	370	117370	5.07	TDF FTC DRV	X	0	X	X
B42	51	454	454	9070	3.96	TDF ABC LPV	X	1	X	X
B43	51	258	290	512000	5.71	AZT 3TC ABC	0	0	0	X
B45	39	460	800	15000	4.18	ABC TDF NVP	0	0	0	X
B46	50	342	342	7313	3.86	ABC 3TC ATV	X	0	X	X
**Mean**	**48.6**	**317**	**391**	**110687**	**4.69**			**1.79**		
**SD**	**12.7**	**180**	**195**	**126507**	**0.66**			**2.14**		
**Median**	**50.0**	**298**	**384**	**84300**	**4.92**			**1.00**		
**1st Quartile**	**42.5**	**201**	**285**	**17218**	**4.23**			**0.00**		
**3rd Quartile**	**52.0**	**375**	**461**	**117013**	**5.07**			**2.25**		

ABC, abacavir; DDI, didanosine; 3TC, lamivudine; TDF, tenofovir; RPV, rilpivirine; AZT, zidovudine; D4T, stavudine; DDC, zalcitabine; FTC, emtricitabine; EFV, efavirenz; NVP, nevirapine; fAPV, fosamprenavir; IDV, indinavir; LPV, lopinavir; NFV, nelfinavir; RTV, ritonavir; SQV, saquinavir; DRV, darunavir

Supplementary Table S2: Biological and virological characteristics of HIV$^+$ patient groups under NNRTI-cART taking into account the last treatment administered (n=23).

Patients	Age (years)	CD4+ Nadir (cells/microl)	CD4+ at initiation of treatment (cells/microl)	HIV RNA Zenith (copies/ml)	HIV RNA Zenith (log copies/ml)	First treatment	PI in the first treatment	Failure of treatment (number)	Always under PI	At least once under PI
**NNRTI at the last treatment (n=23)**										
WSH 227	64	247	247	209000	5.32	AZT 3TC NFV EFV	X	8	0	X
WSH 228	50	300	300	36000	4.56	AZT 3TC NVP	0	4	0	0
WSH 230	34	375	375	/	/	AZT 3TC EFV	0	5	0	X
WSH 242	42	358	619	10200	4.01	AZT 3TC IDV	X	4	0	X
WSH 243	37	369	592	100000	5.00	3TC DDI EFV	0	2	0	0
WSH 244	42	148	133	750000	5.88	AZT 3TC NFV	X	5	0	X
WSH 245	51	170	170	81193	4.91	D4T 3TC IDV	X	4	0	X
WSH 246	52	244	264	50200	4.70	TDF FTC EFV	0	2	0	0
WSH 247	64	204	349	23272	4.37	AZT SQV	X	8	0	X
WSH 248	45	555	555	/	/	3TC ABC	0	2	0	0
WSH 249	44	46	56	750000	5.88	D4T DDI IDV	X	2	0	X
B2	27	292	344	37187	4.57	TDF FTC LPV	X	0	0	X
B3	50	332	332	106000	5.03	TDF FTC APV	X	0	0	X
B6	60	360	360	21000	4.32	AZT 3TC ABC TDF	0	0	0	X
B8	39	975	1000	/	/	EFV FTC TDF	0	0	0	0
B10	66	550	623	/	/	AZT 3TC ABC	0	0	0	X
B14	29	249	265	2072	3.32	TDF FTC LPV	X	0	0	X
B16	53	530	697	82000	4.91	3TC TDF NVP	0	0	0	0
B18	40	70	70	287432	5.46	TDF FTC DRV	X	0	0	X
B21	54	343	343	68000	4.83	ABC TDF NVP	0	0	0	0
B22	40	463	463	194192	5.29	TDF FTC fAPV	X	0	0	X
B29	34	333	410	11500	4.06	AZT 3TC fAPV	X	0	0	X
B37	46	607	607	40900	4.61	AZT 3TC ABC	0	1	0	X
**Mean**	**46.2**	**353**	**399**	**150534**	**4.79**			**2.04**		
**SD**	**10.7**	**197**	**218**	**218335**	**0.62**			**2.53**		
**Median**	**45.0**	**333**	**349**	**68000**	**4.83**			**1.00**		
**1st Quartile**	**39.5**	**246**	**265**	**29636**	**4.47**			**0.00**		
**3rd Quartile**	**52.5**	**419**	**574**	**150096**	**5.16**			**4.00**		
**MW p-value**	**0.495**	**0.579**	**0.903**	**0.892**	**0.892**			**0.985**		

ABC, abacavir; DDI, didanosine; 3TC, lamivudine; TDF, tenofovir; RPV, rilpivirine; AZT, zidovudine; D4T, stavudine; DDC, zalcitabine; FTC, emtricitabine; EFV, efavirenz; NVP, nevirapine; fAPV, fosamprenavir; IDV, indinavir; LPV, lopinavir; NFV, nelfinavir; RTV, ritonavir; SQV, saquinavir; DRV, darunavir
